# *qMrdd2*, a novel quantitative resistance locus for maize rough dwarf disease

**DOI:** 10.1186/s12870-021-03107-1

**Published:** 2021-06-30

**Authors:** Weixiao Zhang, Suining Deng, Yan Zhao, Wei Xu, Qingcai Liu, Yongzhong Zhang, Chunmei Ren, Zhaobang Cheng, Mingliang Xu, Baoshen Liu

**Affiliations:** 1 State Key Laboratory of Crop Biology, Shandong Agricultural University, 61 Daizong Street, Taian, 271018 People’s Republic of China; 2 National Maize Improvement Center, China Agricultural University, 2 West Yuanmingyuan Road, Beijing, 100193 People’s Republic of China; 3 Institute of Plant Protection, Jiangsu Academy of Agricultural Sciences, Nanjing, 210014 People’s Republic of China

**Keywords:** Maize, MRDD, QTL, Fine-mapping, MAS

## Abstract

**Background:**

Maize rough dwarf disease (MRDD), a widespread disease caused by four pathogenic viruses, severely reduces maize yield and grain quality. Resistance against MRDD is a complex trait that controlled by many quantitative trait loci (QTL) and easily influenced by environmental conditions. So far, many studies have reported numbers of resistant QTL, however, only one QTL have been cloned, so it is especially important to map and clone more genes that confer resistance to MRDD.

**Results:**

In the study, a major quantitative trait locus (QTL) *qMrdd2*, which confers resistance to MRDD, was identified and fine mapped. *qMrdd2*, located on chromosome 2, was consistently identified in a 15-Mb interval between the simple sequence repeat (SSR) markers D184 and D1600 by using a recombinant inbred line (RIL) population derived from a cross between resistant (“80007”) and susceptible (“80044”) inbred lines. Using a recombinant-derived progeny test strategy, *qMrdd2* was delineated to an interval of 577 kb flanked by markers N31 and N42. We further demonstrated that *qMrdd2* is an incompletely dominant resistance locus for MRDD that reduced the disease severity index by 20.4%.

**Conclusions:**

A major resistance QTL (*qMrdd2*) have been identified and successfully refined into 577 kb region. This locus will be valuable for improving maize variety resistance to MRDD via marker-assisted selection (MAS).

**Supplementary Information:**

The online version contains supplementary material available at 10.1186/s12870-021-03107-1.

## Background

Maize rough dwarf disease (MRDD) is a serious viral disease worldwide. MRDD is caused by four pathogenic viruses [1], namely, Maize rough dwarf virus (MRDV), Mal de Rio Cuarto virus (MRCV), Rice black-streaked dwarf virus (RBSDV) and Southern rice black-streaked dwarf virus (SRBSDV), which are prevalent in Europe [2], South America [3], Asia [4], and Northern China [5], respectively. These pathogenic viruses are naturally transmitted by the small brown planthopper *Laodelphax striatellus* in a persistent manner [6] and are not spread by seed or mechanical inoculation. Once infected, *L. striatellus* can transmit these viruses for life, though transmission to eggs does not occur [7].

MRDD causes substantial yield losses in maize production globally [2]. Indeed, between 2008 and 2011, MRDD was responsible for yield losses among more than 3,000,000 ha of crops each year in North China with yield losses reaching 100% in some seriously affected areas [8]. The disease leads to severe symptoms, such as plant dwarfing, internode shortening, waxy enation, malformed tassels and dark-green leaves [9]. Current methods for controlling MRDD are agricultural measures (i.e., delayed sowing) and chemical control. However, delayed sowing leads to the underutilization of photothermal resources and renders the seedlings vulnerable to waterlogging and late ripening, as occurred in the Yellow-Huai-Hai River plain [10]. Small-scale chemical control is also not ideal and leads to environmental pollution. Accordingly, cloning natural resistance genes and breeding resistant maize varieties would be the most environmentally responsible and cost-effective means of controlling MRDD. Hence, collecting and identifying various resistant germplasms will facilitate the development of resistant maize cultivars, and it is especially important to map and clone resistant genes to MRDD.

Most research indicates that maize resistance to MRDD is controlled by many genes, each with different effects [2, 11, 12]. Maize germplasms shows different levels of resistance to MRDD under natural conditions, with the most resistant germplasm originating from US hybrid P78599 [13–16]. By using F_3_ families of Mo17 and BSL14, Di Renzo et al. [17] found that in Argentina, the heritability of MRCV resistance in maize ranged from 0.44 to 0.56, and based on an F_2:3_ QTL-mapping strategy, 2 quantitative trait loci (QTLs) were localized to maize chromosome bins 1.03 and 8.03/4 [18]. Luan et al. [19] screened four linked molecular markers and mapped three QTLs for resistance to MRDD within chromosome bins 6.02, 7.02 and 8.07 by using an F_2_ population derived from the resistant inbred line 90,110 and the susceptible inbred line Ye478. Through association analysis with 163 inbred lines, Shi detected one associated single nucleotide polymorphism (SNP) in the *ZmeIF4E* promoter, which accounting for 3.33 and 9.04% of the phenotypic variation, respectively in two environments, [20]. Using 152 maize germplasm isolates and 89 recombinant inbred lines (RILs) derived from resistant line X178 and susceptible line B73, Shi et al. [21] detected three QTLs for MRDD resistance in bins 2.07, 5.04 and 8.03, and the QTL on bin 8.03 explained 24.6–37.3% of the phenotypic variance. This major QTL (*qMrdd8*) on chromosome bin 8.03 was then fine-mapped to a 347-kb region by Shi’s laboratory [22]. With 527 inbred lines and 556,000 SNPs, 15 candidate genes associated with MRDD resistance have been identified [23]. Conventional linkage and high-throughput sequencing analysis have been employed to locate the resistance-related genomic region, and an associated region was identified within the genomic interval 68,396,487 − 69,523,478 bp on chromosome 6 [24]. A major QTL (*qMrdd1*) that reduces the DSI by 24.2–39.3% was fine-mapped to a 1.2-Mb region by applying recombinant-derived progeny testing to self-pollinated backcrossed families [25]. Although many QTLs have been identified, few have been fine-mapped or even cloned. Liu et al. (2020) reported that the Rab GDP dissociation inhibitor *(ZmGDIα*) is a candidate gene for *qMrdd1* which confers recessive resistance to MRDD and resolves the underlying molecular mechanisms controlling viral diseases. *qMrdd1* is also the only resistance gene cloned to date [26].

Considering the damage caused by MRDD and the complexity of disease resistance, it is critical to identify new resistance QTLs that can be used to genetically control MRDD and to deepen our understanding of resistance mechanisms.

## Results

### Initial QTL analysis of MRDD

**Fig. 1:**
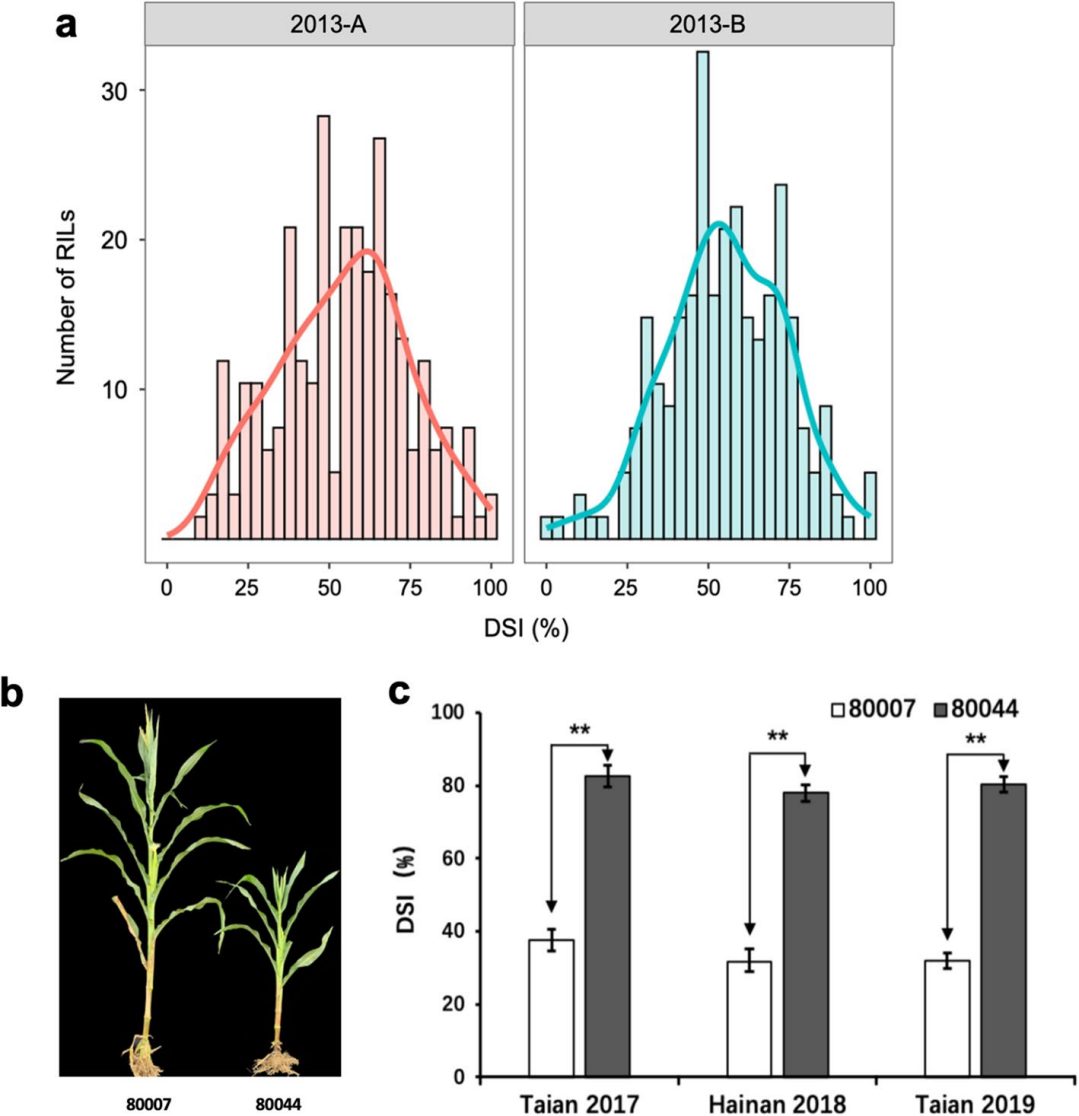
The phenotype of MRDD in the RILs and parental lines. **a** Frequency distribution of DSI (%) in the RIL population in two field trials (2013-A and 2013-B). The number of individuals in each phenotypic class is indicated on the *y-axis* and the phenotypic score classes on the *x-axis*. **b** Individual plants from parental lines “80007” and “80044” after artificial inoculation. **c** The DSI of the parental lines in Taian in 2017, Hainan in 2018 and Taian in 2019 (**P < 0.01; two-tailed Student’s t-test)

For each field trial, the RIL population showed continuous variation in DSI (range 0–100%), with averages of 54.2 and 55.5% in two field trials (2013-A and 2013-B) respectively (Fig. 1a). Spearman’s rank correlation coefficient (*r*_*s*_) among two sets of phenotypic data was calculated. The data among two replications were significantly correlated (p-value = 6.74E^−13^) with *r*_*s*_ was 0.49. The broad-sense heritability (*H*^*2*^*)* of disease resistance was estimated to be 0.66 (Table S1), and the variance was mainly derived from differences among RILs (Table 1). These results suggested that the genetic factor plays a major role in maize resistance to MRDD within the RIL population.

**Table 1:** Analysis of variance of data in the RIL population

**Sources**	***df***	**SS**	**MS**	***F-*value**	***P-*value**	**Variance**
RILs	199	65.53	0.29	8.67	3.66E-76***	1.35E-03
Environments	2	2.17	1.13	76.0	8.23E-26 ***	5.16E-03
Residuals	398	17.3	3.16E-03			2.15E-03

*Sources* Variation sources, *df* Degrees of freedom, *SS* Sum of squares, *MS* Mean squares, *P-value* Significant difference among sources

^***^P < 0.001

Two maize lines, “80007” and “80044”, were evaluated for resistance to MRDD under artificial inoculation 3 times: in 2017 and 2019 in Taian and in 2018 in Hainan. For each field trial, line “80007” showed effective resistance to MRDD, whereas line “80044” was highly susceptible; the average DSIs of “80007” and “80044” were 34.6 and 82.03%, respectively (Fig. 1b, c).

### Initial QTL mapping of MRDD resistance

Based on 199 RILs and 804 informative SNPs, we constructed a genetic linkage map that covered 1291.05 cM, with an average of 1.61 cM between adjacent markers (Fig. S1, Fig. S2, Table S2 and Table S3). The DSI for the 199 RILs was used as phenotypic data for QTL mapping of MRDD resistance. Two QTLs were consistently detected in all three field trials (2013-A, 2013-B and the average DSI phenotype of each RILs in 2013-A and 2013-B) (Fig. S3, Fig. S4 and Table 2). One of the QTLs, *qMrdd2*, is located on chromosome 2 within a 15-Mb region and explained 12.88 − 15.67% of the total phenotypic variation. Another QTL, located on chromosome 5, explained ~ 6% of the total phenotypic variation (Table 2).

**Table 2:** The QTL related to MRDD in three field trials

Field trial	Chromosome	Position (Mb)	LOD	Additive effect	SRA	*R*^2^
Average	2	15.10 − 39.30	5.5581	-0.0503	80007	0.1156
	3	20.42–27.67	3.3725	-0.0397	80007	0.0730
	5	50.90 − 70.26	3.8923	-0.0446	80007	0.0625
	8	200.13–219.86	2.8755	-0.0350	80007	0.0566
	10	0.44–16.38	3.1656	-0.0370	80007	0.0617
R 1	2	14.80 − 37.48	5.1172	-0.0675	80007	0.1588
	5	54.55 − 70.26	2.9262	-0.0425	80007	0.0614
R 2	2	13.41 − 37.48	4.8036	-0.0648	80007	0.1288
	3	20.42–27.67	3.1145	-0.0391	80007	0.0728
	5	50.89 − 70.26	2.8259	-0.0462	80007	0.0641

*LOD* Logarithm of odds, *SRA* Source of resistance allele, *R*^*2*^ Explained phenotypic variation; “R1” and “R2” represent the field trial 2013-A and 2013-B and “Average” is the average value of 2013-A and 2013-B

### Fine mapping of *qMrdd2*

According to the initial QTL analysis, two flanking markers (D184 and D1600) and one marker residing within the *qMrdd2* region (D829-0) were used to screen the RIL population. Ultimately, six recombinant RILs that had crossover breakpoints within the *qMrdd2* region were detected and further genotyped with seven newly isolated markers (D387-2, D550, D664, D829-0, D936-2, D122-6 and D1347) (Table S4). All six F_5_ recombinants were self-pollinated to produce corresponding F_6_ progeny. In the summer of 2017, the F_6_ progeny (1010 plants) were planted in Taian to test for MRDD resistance in the field. Three genotypes within each F_6_ progeny derived from individual heterozygous recombinant plants were categorized: *qMrdd2* (80007) homozygote and heterozygote and nonq*Mrdd2* (80044) homozygote. The DSI value for each genotype was calculated. MRDD resistance among the three genotypes of recombinant types a-I, a-III, a-IV, a-V and a-VI was not significantly different (*P* < 0.05), indicating that *qMrdd2* is located in the homozygous region of the parental region. For the remaining recombinant type a-II, a statistically significant difference (*P* < 0.05) in MRDD resistance among the three genotypes was observed, suggesting the presence of *qMrdd2* in the heterozygous region of the parental recombinant. This analysis shows that the *qMrdd2* locus was localized to between markers D829-0 and D122-6 with a physical distance of ~ 3.9 Mb based on the B73 physical map (AGPv4).

**Fig. 2:**
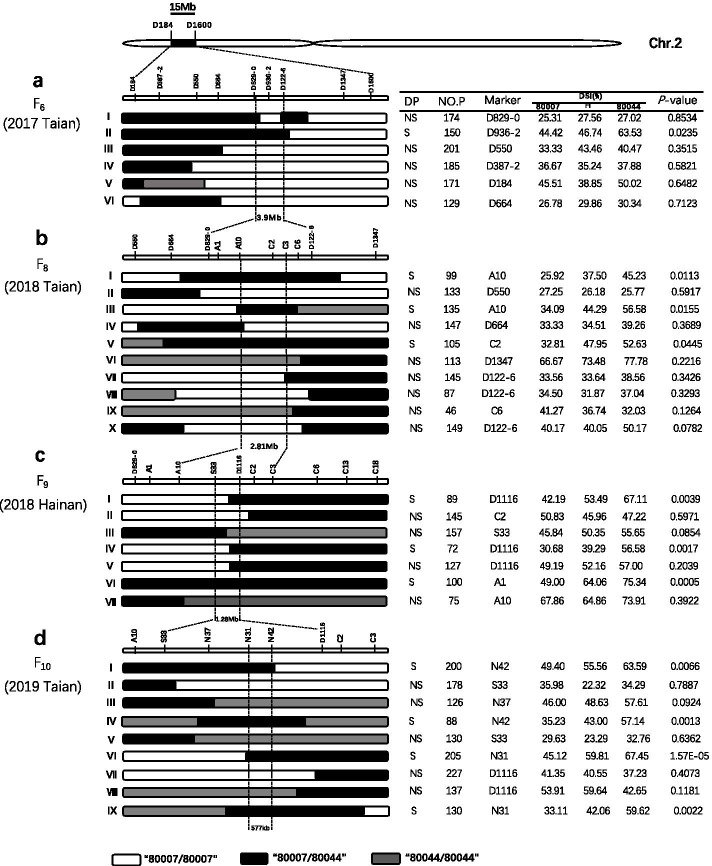
Sequential fine-mapping of the major QTL *qMrdd2* in maize. Six F_6_ (**a**), 10 F_8_ (**b**), 7 F_9_ (**c**) and 9 F_10_ (**d**) recombinants and their corresponding recombination types are shown. For each recombinant type, the chromosomal composition at *qMrdd2* is shown as black, white or gray rectangles, corresponding to heterozygous “80007”/”80044”, homozygous “80007”/”80007” and homozygous “80044”/”80044”, respectively. All progeny were genotyped using markers within the heterozygous “80007”/”80044” segment. The DSI value was calculated for each plant of the three genotypes. A significant difference (*P* < 0.05) in DSI between homozygous “80007”/”80007” and homozygous “80044”/”80044” indicated the presence of *qMrdd2* in the heterozygous region of their parental recombinant(s) and that the recombinant(s) was segregating (S). However, this was not the case between the two homozygous genotypes, suggesting the absence of *qMrdd2* in the heterozygous region and that their parental recombinant(s) was not segregating (NS). Based on analysis of both genotype and phenotype for all recombinant types, the map of *qMrdd2* was refined from an ~ 15-Mb region to an ~ 577-kb region flanked by markers S31 and S42. DP: deduced phenotype, No. P: number of recombinant plant progeny, “80007”: progeny with a homozygous “80007” genotype, H: progeny with a heterozygous “80007”/”80044” genotype, “80044”: progeny with a homozygous “80044” genotype

In the winter of 2017 in Hainan, nine F_7_ progeny (603 plants) were planted in Hainan, and five InDel markers (A1, A10, C2, C3 and C6) were designed to genotype new recombinants. Ten types of recombinants were identified and self-pollinated to produce 10 F_8_ families (1159 plants) that were planted in Taian in 2018. Ten markers (D550, D664, D829-0, A1, A10, C2, C3, C6, D122-6 and D1347) were used to genotype the 10 types of recombinants (I–X; Fig. 2b). Based on the statistically significant difference (*P* < 0.05) in DSI value between “80007” and “80044” homozygotes, recombinants b-I, b-III, and b-V were considered to be resistant to MRDD (Fig. 2b), suggesting that *qMrdd2* is located in the heterozygous region of these recombinant types. In contrast, recombinant types b-II, b-IV, and b-VI − b-X did not show a statistically significant difference (*P* > 0.05) in DSI between “80007” and “80044” homozygotes (Fig. 2b), indicating that *qMrdd2* is in the homozygous region. Thus, *qMrdd2* was localized to the 2.81-Mb (AGPv4) (http://www.maizegdb.org) interval between markers A10 and C3 (Fig. 2b).

Seven types of recombinants were identified with markers A10 and C3 from the F_8_ progeny which were then self-pollinated to generate 7 F_9_ families (765 plants). All F_9_ progeny were planted in Hainan Province in the winter of 2018. Three markers (S33, D1116 and C2) in the mapped A10/C3 interval and another seven newly developed markers (D829-0, A1, A10, C3, C6, C13 and C18) were applied to genotype the 7 recombinants (Fig. 2c). The same progeny test strategy was utilized to determine whether there was a statistically significant difference (*P* < 0.05) in DSI value between “80007” and “80044” homozygotes. The results for type c-I, c-IV and c-VI recombinants indicated that *qMrdd2* is located in the heterozygous region. Analogously, *qMrdd2* was localized to the homozygous region of type c-II, c-III, c-V and c-VII recombinants (Fig. 2c). Therefore, *qMrdd2* was mapped between markers S33 and D1116 with a physical distance of 1.28-Mb (AGPv4).

We conducted the fourth fine-mapping in the summer of 2019 in Taian. Three InDel markers (N37, N31 and N42) were developed in the newly mapped 1.28-Mb region, and 1421 individuals from 9 recombinants were obtained from F_9_ progeny. The progeny of types d-I, d-IV, d-VI and d-IX show significantly (*P* < 0.05) different DSI values, and types d-II, d-III, d-V, d-VII and d-VIII did not show significant differences (*P* > 0.05) in DSI. These data further allowed us to localize *qMrdd2* to the 577-kb (AGPv4) interval flanked by markers N31 and N42 (Fig. 2d).

### Genetic model of *qMrdd2* resistance to MRDD

**Fig. 3:**
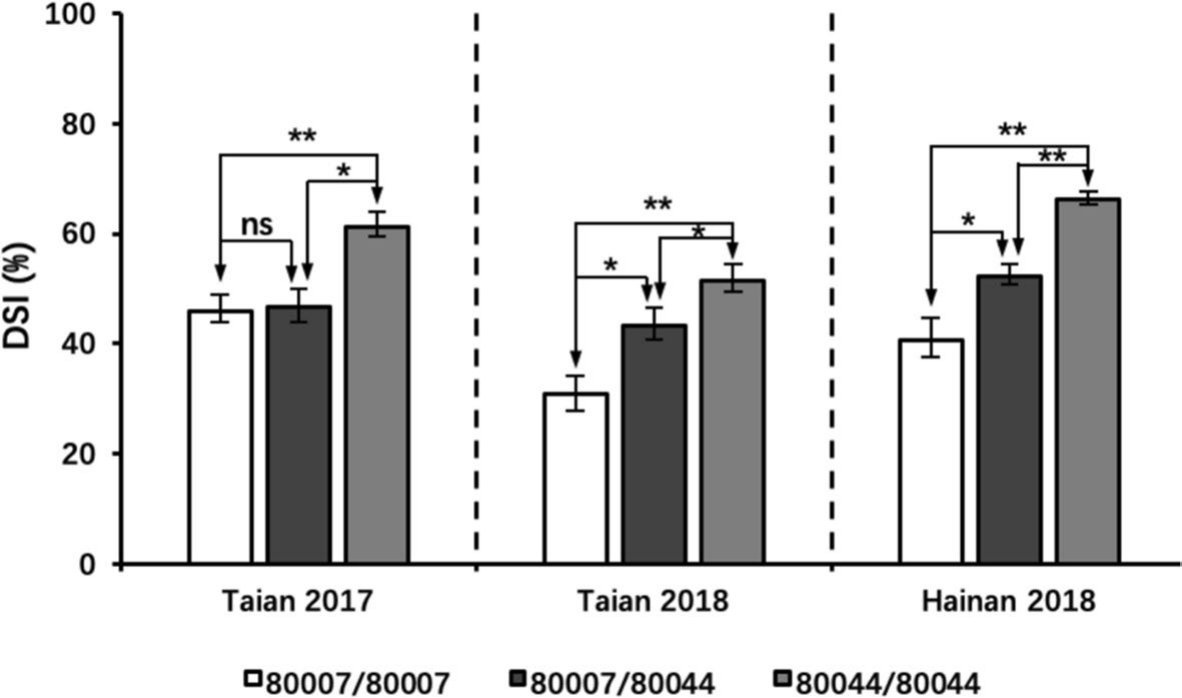
Genetic effect of the QTL *qMrdd2* on F_n_ populations across three experimental sites. F_n_ populations were divided into three genotypes (“80007”/”80007”, “80007”/”80044” and “80044”/”80044”) according to genotypes within the *qMrdd2* region. Average DSI values are shown (*P < 0.05; **P < 0.01; ns, not significant; two-tailed Student’s *t*-test)

F_6_, F_8_ and F_9_ families were used to investigate the genetic effect of *qMrdd2*. Plants with homozygous “80007”/”80007”, heterozygous “80007”/”80044” and homozygous “80044”/”80044” genotypes showed DSIs of 30.94, 43.35 and 51.48% in 2017 Taian, 30.94, 43.35 and 51.48% in 2018 Taian, 40.62, 52.28 and 66.23% in 2018 Hainan, respectively. For each field trial, homozygous “80044”/”80044” plants showed an obvious difference in DSI relative to homozygous “80007”/”80007” plants, with an average difference of 20.4%. The DSI of heterozygous plants was, on average, 12.17% greater than that of homozygous “80007”/”80007” plants (Fig. 3). These results indicated that the *qMrdd2* QTL acts in an incompletely dominant manner to confer resistance to MRDD.

## Discussion

The recombinant-derived progeny test is an efficient method and usually used to detect a QTL in backcrossed population such as *qRfg2* and *qhmf4* [27–29]. Compared with backcrossed progeny, the self-pollinated progeny can capture the effect of all three genotypes and create more recombinants for fine- mapping. However, if recombinants are homozygous at both flanking markers, they produced by self-pollination cannot be used for fine-mapping. Crosses with other heterozygous plants in the same family can solve this problem very effective.

An artificial infection process [30] was used to evaluate the MRDD resistance of individual plants in this study, in which adequate viruliferous planthoppers carrying RBSDV provided stable and uniform disease pressure. In 2017 in Hainan, because the seedling age was inconsistent with the planthoppers, the planthoppers were old with low motility when the seed germinates. As a result, planthopper activity was inadequate to assess most of the recombinant individual phenotypes. In the spring of 2019, disease-resistant phenotypes in the F_9_ population were not as robust as those in previous experiments, which may have resulted from a severe cold snap during the seedling stage. As the first survey of disease symptoms was conducted at 40 days after inoculation, the most severely affected plants could be analyzed before they died, followed by second and third surveys after pollination at 10-day intervals to ensure consistency of the phenotypic data.

We previously identified a major resistance QTL (*qMrdd1* on chr.8) from the highly resistant inbred line NT411 and X178 [25]. Additional resistance QTLs have been identified on chr.1, 2, 5, 6, and 7 [19, 24, 31, 32]. In this study, using a different resistant inbred line (80007), we detected another major QTL (*qMrdd2*, chr.2, bin 2.01/02) and localized it to an interval of ~ 577 kb. A resistance QTL, previously mapped on chr.2 (bin 2.07/08) [8] is clearly different from *qMrdd2* (bin 2.01/02). Also, *qMrdd2* could be validated in the context of association mapping, by exploring a panel of accessions which usually contains a great diversity from one or multiple germplasms.

In this study, the *qMrdd2* was narrowed down to an ~ 577 kb region (APV 4). Gene prediction by FEGNESH 2.6 (http://linux1.softberry.com/berry.phtml) has revealed 29 putative genes in the *qMrdd2* region. Most of the genes have not been annotated yet, only one (*Zm00001d002312*) is related to disease resistance. The gene *Zm00001d002312* was annotated as Leucine-rich repeat transmembrane protein kinase (LRR-TM) in the MazieGDB and the LRR-TM genes are important receptor resistance genes in plants in response to pathogen infection [33]. Both *Cf-2* and *HcrVf2* were this type of gene, which were related to the resistance to *Cladosporium fulvum* in tomato and *Scab* in apple [34, 35], respectively. While *Zm00001d002312* is a good candidate gene for *qMrdd2*, further experiments such as genetic mapping by screening new recombinants within the *qMrdd2* locus or gene expression and transgenic testing studies must be performed to restrict the *qMrdd2* locus to a single gene.

Because *qMrdd2* is an incompletely dominant resistance QTL, the *qMrdd2* donor region should be integrated into both parental lines via MAS to guarantee maximally resistant F_1_ hybrids. However, given that homozygous segments from both parental lines would impair heterosis, it is important to minimize the overlapped *qMrdd2* region in the F_1_ hybrid for successful molecular breeding. We propose that one parental line carry the shortest possible *qMrdd2* donor region on the left, while the other parental line carry the shortest possible *qMrdd2* donor region on the right via MAS to minimize such overlap. Therefore, developing suitable markers to identify the shortest possible *qMrdd2* donor region to minimize such overlap is of great importance. In present study, the genetic distance between marker N31 and N42 was ~ 0.67 cM. Thus, they may be effective for MAS of MRDD resistance because this marker is tightly linked within 5 cM of the QTL [36].

## Conclusions

It is critical to identify new resistance QTLs that can be used to genetically control MRDD and to deepen our understanding of resistance mechanisms. With the availability of a RIL population, we here detected a number of consistent MRDD QTL. Using a recombinant-derived progeny test strategy, A major resistance QTL (*qMrdd2*) have been identified and successfully refined into 577 kb region. We further demonstrated that *qMrdd2* is an incompletely dominant resistance locus for MRDD that reduced the disease severity index by 20.4%. This locus will be valuable for improving maize variety resistance to MRDD via MAS.

## Methods

### Plant materials

**Fig. 4:**
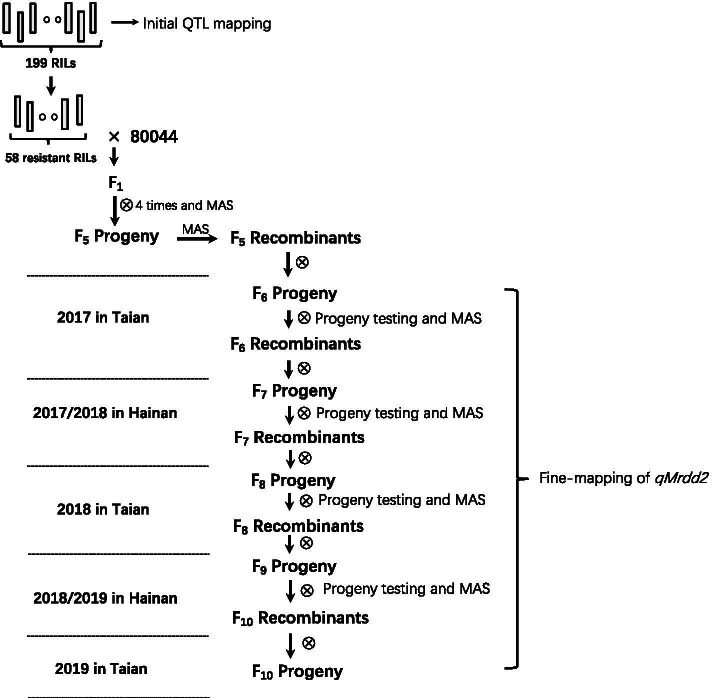
Experimental flow chart for QTL identification and fine-mapping of QTL *qMrdd2*, which confers resistance to MRDD. A total of 199 RILs were used for the initial QTL mapping. Recombinants identified within the F_6_-F_9_ populations were genotyped and investigated to fine-map the QTL *qMrdd2*

An RIL population consisting of 199 lines developed by single-seed descent from a cross between the inbred lines “80044” and “80007” was used in this study. The “80044” is a susceptible line bred from a Reid heterotic group, and “80007” is a resistant line from the P heterotic group. The two parents as well as the RILs were all developed by Prof. Baoshen Liu of Shandong Agricultural University. For the initial QTL mapping in 2013, we planted 199 RILs as the initial QTL mapping population for conducting two field trials (2013-A and 2013-B) at an experimental station (Jining, Shandong Province). For each RIL, 30 seeds were sown in one row with a spacing of 0.6 m between rows and 0.25 m between plants within a row. For fine mapping, 58 resistant RILs with different chromosomal recombinations in the QTL region were chosen and backcrossed with the susceptible line “80044” to produce F_1_ populations. Using marker-assisted selection (MAS), the F_1_ hybrids with the target QTL were self-pollinated four times to produce an F_5_ population; all mapping populations were developed based on the experimental flow chart presented in Fig. 4. All populations were grown at the experimental station of Shandong Agricultural University (Tai'an, Shandong Province) and experimental base (Sanya, Hainan Province) for fine mapping. For comparison, we planted two parents in each fine mapping, all populations and two parent lines were artificially inoculated with viruliferous planthoppers (see below).

Molecular markers within the major QTL region were used to identify recombinants from the F_5_ population, and the recombinants were self-pollinated to generate F_6_ progeny. This process was repeated during 2017–2019 to develop a series of advanced self-pollinated populations consisting of 1010 F_6_, 603 F_7_, 1159 F_8_, 765 F_9_ and 1421 F_10_ plants for fine mapping of *qMrdd2*. MAS and progeny testing were conducted across self-pollinated populations until fine mapping of the target QTL was completed.

### Artificial inoculation and scoring of plants for symptoms

Planthoppers carrying RBSDV were provided by Jiangsu Academy of Agricultural Sciences (Nanjing, China) [37]. Before inoculation, maize kernels derived from one recombinant plant were placed in a plastic casing, and a Dot-ELISA [38] was used to detect the infection rate of the planthoppers. Seedlings inoculated at the coleoptile stage with one infected planthopper [39] were cultured at 24 °C in a greenhouse for 72 h; the seedlings were planted in a sample plot with 0.3-m spacing between plants and 0.6-m spacing between rows.

**Fig. 5:**
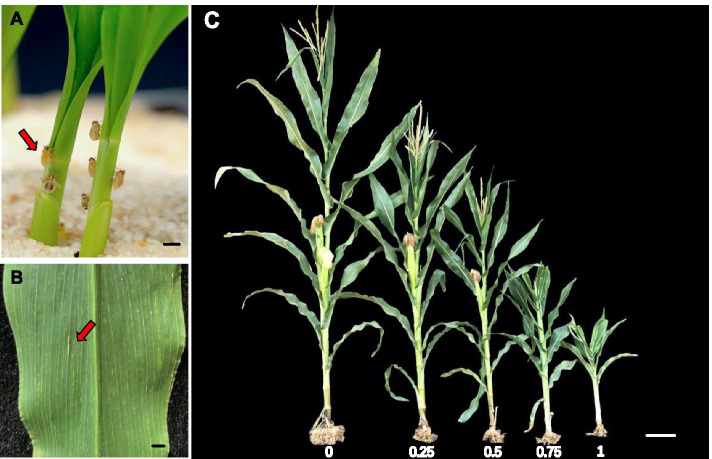
Symptom of RBSDV on *Zea mays* L. **A** Rice black-streaked dwarf virus (RBSDV) is transmitted to its natural host, maize, by the brown planthopper (*Laodelphax striatellus*). Bar = 5 mm. **B** Waxy enations on abaxial surfaces of leaves present in maize plants infected with RBSDV. Bar = 5 mm. **C** Maize with MRDD scores of 0–1. Disease scores were primarily based on plant height. Bar = 10 cm

Symptom scoring was conducted at 40 days after inoculation and was repeated twice after pollination at 10-day intervals. For QTL analysis and fine-mapping, a scoring system (0, 0.25, 0.5, 0.75 or 1) based on overall symptoms was adopted to evaluate MRDD resistance (Fig. 5), as follows: 0 = no symptoms; 0.25 = slightly shortened superior internodes, resulting in plants that are ~ 80% of the height of a healthy plant; 0.5 = dark-green leaves, waxy enations on abaxial surfaces of leaves and sheaths and obviously shortened superior internodes, resulting in plants that are ~ 66% of the height of a healthy plant; 0.75 = severely shortened internodes and malformed tassels, resulting in plants that are ~ 50% of the height of a healthy plant; 1 = severe stunting, with suppressed flowering and the absence of ears, resulting in plants that are < 50% of the height of a healthy plant. DSI was calculated by the following formula: DSI (%) = ∑ [(scale × number of plants in scale) / (1 × total number of plants)] × 100%, and was used to represent the MRDD severity of families [40].

### Analysis of phenotypic data

Statistical analysis was performed using R project [39]. Two sets of phenotypic data (2013-A, 2013-B) were collected for the RIL population and the Spearman’s rank correlation coefficient (*r*_*s*_) of them was calculated. We used ANOVA (part of the R project) to test the significance of the genotypic and environmental effects. The model for variance analysis is as follows: *y*_*i*_ = *μ* + *gi* + *ej* + *ε*, in which *y*_*i*_ is the phenotypic value of the *i*th RIL line, *μ* is the overall average value, *gi* the genetic effect of *i*th RIL, *ej* the environmental effect of *j*th trial and *ε* is the residual. The ANOVA result was used to calculate the broad-sense heritability (*H*^*2*^ = *σ*_*g*_^2^/ *σ*_*g*_^2^ + *σ*_*ε*_^2^/*e*), where *σ*_*g*_^2^ is the genetic variance, *σ*_*ε*_^2^is the residual error, and *e* is the number of environments [41, 42].

Student’s t-test was performed in QTL fine-mapping study to compare the difference in DSI between the two parent lines “80007” and “80044” and the three genotypes (“80007”/”80007”, “80007”/”80044” and “80044”/”80044”) in self-pollinated populations.

### Linkage map construction and detection of QTLs for resistance to MRDD

Genotype data for the RIL population were collected using an Illumina Golden Gate 3 K SNP chip. We obtained 3072 SNP markers from Illumina Golden Gate 3 K SNP chip and six criteria were applied to analysis SNP database (Table S5). Based on these genotype data, we employed JoinMap (Version 2.5) [43] to construct a genetic linkage map and the Kosambi mapping function [44] to calculate the genetic distances. QTL analysis was carried out in Windows QTL Cartographer (Version 2.5) [45] with the composite interval mapping method [46], a walk speed of 2.0 cM and a window size of 10 cM. For each of the datasets (R1, R2, and Average), a significance threshold to confirm a putative QTL was obtained from a 1000-permutation test at P < 0.05 with a logarithm of the odds (LOD) score > 2.5 [47].

### Genotyping

Leaf tissue from each plant in the field was collected at the six- to seven-leaf stage. An SDS procedure [48] was used to extract genomic DNA. Maize insertion or deletion (InDel) and SSR markers were retrieved from MaizeGDB (http://www.maizegdb.org/); the primers were produced by Sangon Biotech (Shanghai, China). Each sample was subjected to genotyping using the markers as required, and PCR products were separated by 1% agarose gel or 6% polyacrylamide gel electrophoresis.

### Development of PCR-based markers

Based on the initial QTL analysis, *qMrdd2* was mapped to the confidence region between D184 and D1600, which covers a physical distance of 15 Mb based on the B73 physical map (AGPv4). Therefore, we saturated the region with high-density molecular markers. Two types of markers were developed based on different sequence variations, including SSRs and InDels. To develop SSR markers, the maize B73 reference sequence in the QTL region was downloaded from the website http://plants.ensembl.org/Zea_mays/Info/Index/. The sequences were scanned by the software SSRHunter (Version 1.3) to search for SSRs, and primers flanking the SSRs were designed with the PrimerQuest tool (https://sg.idtdna.com/Primerquest/Home/Index). To develop InDel markers, lines “80007” and “80044” were sequenced by Tsingke Biotech (Beijing, China), and InDels in the 15 Mb interval for the QTL were analyzed. Single-/low-copy sequences were detected by BLAST on MaizeGDB with the B73 genomic sequence database. BLAST results were surveyed for InDels of ≥ 3 bp, which were detected as 3-bp gaps in the alignment of sequences from the two parents. InDels with ≥ 1000 bp of upstream sequence and ≥ 1000 bp of downstream sequence were assessed by PCR. Primers were designed by Primers Input (https://primer3.ut.ee) with the targets (970,60). All primers met to the following criteria: ~ 20 nucleotides with 40–60% GC content, no consecutive tracts of a single nucleotide and no secondary structure. Ultimately, 21 markers (10 SSRs and 11 InDels) were found to be polymorphic between the parental lines (Table S4).

### Strategy for fine-mapping of *qMrdd2*

Appropriate marker density, crossover density and the ability to determine the precise phenotype of each recombinant are essential for fine mapping analysis. Based on the initial QTL mapping, we developed high-density markers within the 15-Mb interval of *qMrdd2*. Sequential fine mapping of *qMrdd2* was carried out by using recombinant-derived progeny [27]. In this study, a robust progeny test strategy was employed to evaluate the MRDD phenotype of all recombinants (Fig. 4). The DSI was used to represent MRDD severity, as previously described in detail (Fig. 5). Recombinants developed from the F_n_ population were self-pollinated, and the resulting self-pollinated population was grown in the same experimental stations under artificial infection. Each individual was genotyped using markers in the heterozygous region of its parental recombinant and scored for disease severity in the field. A DSI value was estimated for each resulting self-pollinated population. The progeny from each recombinant strain were divided into three genotypes: “80007” homozygote, “80044” homozygote and “80007”/”80044” heterozygote. Statistically significant differences in the DSI value of each genotype were determined using two-tailed Student’s *t*-test. If there was a significant difference (*P* < 0.05) in DSI value between the two homozygous genotypes, *qMrdd2* was considered to be located in the ‘80007’ donor segment, namely, in the heterozygous region of the parental recombinant. If there was no significant difference (*P* ≥ 0.05) in DSI value between the two homozygous genotypes, *qMrdd2* was assumed to be absent in the ‘80007’ donor segment, namely, in the homozygous region of the parental recombinant, or no *qMrdd2* was present.

## Supplementary Information

**Additional file 1: Table S1.** Spearman’s rank correlation coefficient (*r*_*s*_*)* of DSI between the two field trials. **Table S2.** Marker and genetic distance information for the 10 maize linkage groups. **Fig. S1.** The ten maize genetic linkage groups. **Fig. S2.** Linkage disequilibrium (LD) heat map of each linkage group. **Table S3.** The number of SNP markers accepted as cofactors in the statistical model of QTL mapping. **Fig. S3.** Detection of QTLs conferring resistance to MRDD. **Fig. S4.** Boxplot graph of DSI values of the initial mapping population. **Table S4.** Markers developed to map the *qMrdd2* locus. **Table S5.** Criteria applied to analysis SNP database.

## Data Availability

The sequencing data is available from the corresponding author on reasonable request. Other data produced during this study are included in our article and in its supplementary files.

## Abbreviations

MRDD: Maize rough dwarf disease
QTL: Quantitative trait loci
SSR: Simple sequence repeat
RIL: Recombinant inbred line
MAS: Marker-assisted selection
MRDV: Maize rough dwarf virus
MRCV: Mal de Rio Cuarto virus
SRBSDV: Southern rice black-streaked dwarf virus
InDel: Insertion or deletion
