# Supplementary material for: qMrdd2, a novel quantitative resistance locus for maize rough dwarf disease
Source: BMC Plant Biol. 2021 Jun 30;21:307. doi: 10.1186/s12870-021-03107-1 (PMC8244169; doi:10.1186/s12870-021-03107-1)
Supplement: Supplementary file 1 — Additional file 1: Table S1. Spearman’s rank correlation coefficient (rs) of DSI between the two field trials. Table S2. Marker and genetic distance information for the 10 maize linkage groups. Fig. S1. The ten maize genetic linkage groups. Fig. S2. Linkage disequilibrium (LD) heat map of each linkage group. Table S3. The number of SNP markers accepted as cofactors in the statistical model of QTL mapping. Fig. S3. Detection of QTLs conferring resistance to MRDD. Fig. S4. Boxplot graph of DSI values of the initial mapping population. Table S4. Markers developed to map the qMrdd2 locus. Table S5. Criteria applied to analysis SNP database. [file 12870_2021_3107_MOESM1_ESM.docx]

**Table S1** Spearman’s rank correlation coefficient (*r_s_)* of DSI between the two field trials

|  | 2013-A | 2013-B |
| --- | --- | --- |
| 2013-A | 1 |  |
| 2013-B | 6.74E^-13^ ** | 1 |

The values in the table indicate the *r_s_* and its significant difference: **P< 0.01.

**Table S2** Marker and genetic distance information for the 10 maize linkage groups

| **Chromosome** | | **No. of markers** | **Genetic distance (cM)** |
| --- | --- | --- | --- |
| 1 | 107 | 206.06 |  |
| 2 | 84 | 153.54 |  |
| 3 | 86 | 129.56 |  |
| 4 | 114 | 148.76 |  |
| 5 | 76 | 138.07 |  |
| 6 | 83 | 110.45 |  |
| 7 | 84 | 91.37 |  |
| 8 | 63 | 141.73 |  |
| 9 | 53 | 87.93 |  |
| 10 | 54 | 83.58 |  |
| Total | 804 | 1291.05 |  |

No. of markers: number of markers on each chromosome.


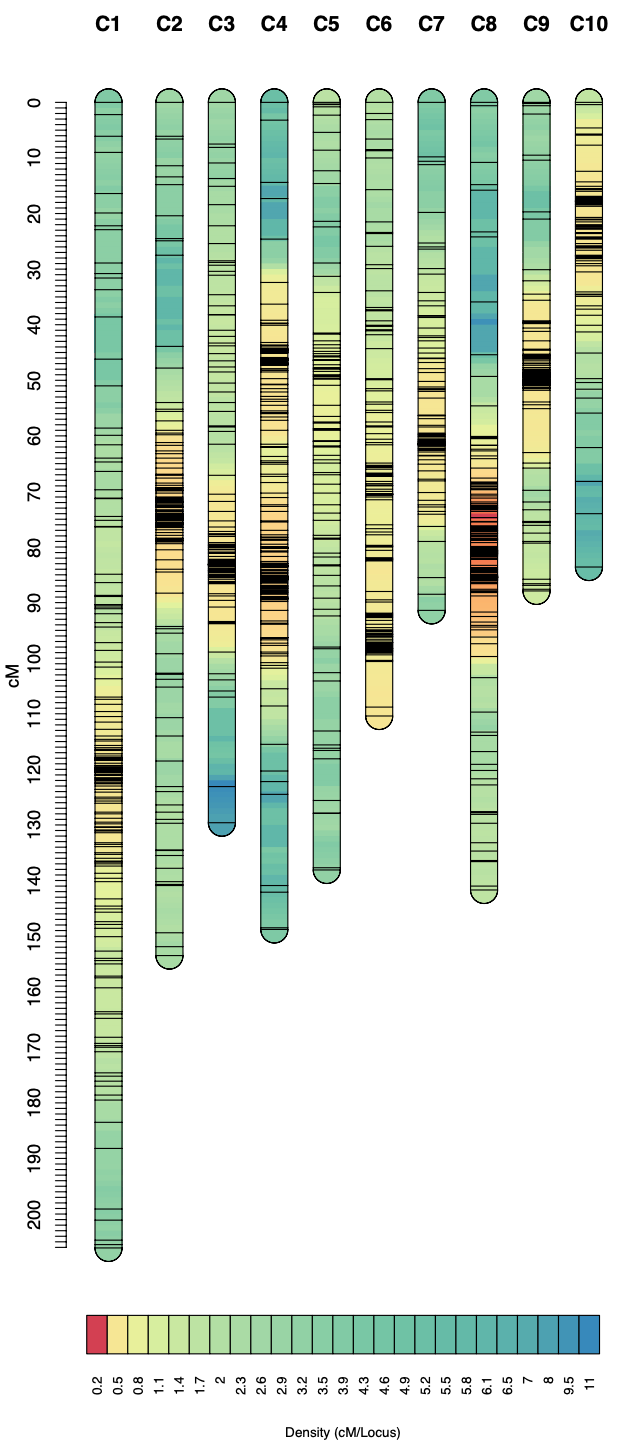


**Fig. S1** The ten maize genetic linkage groups. C: chromosome.


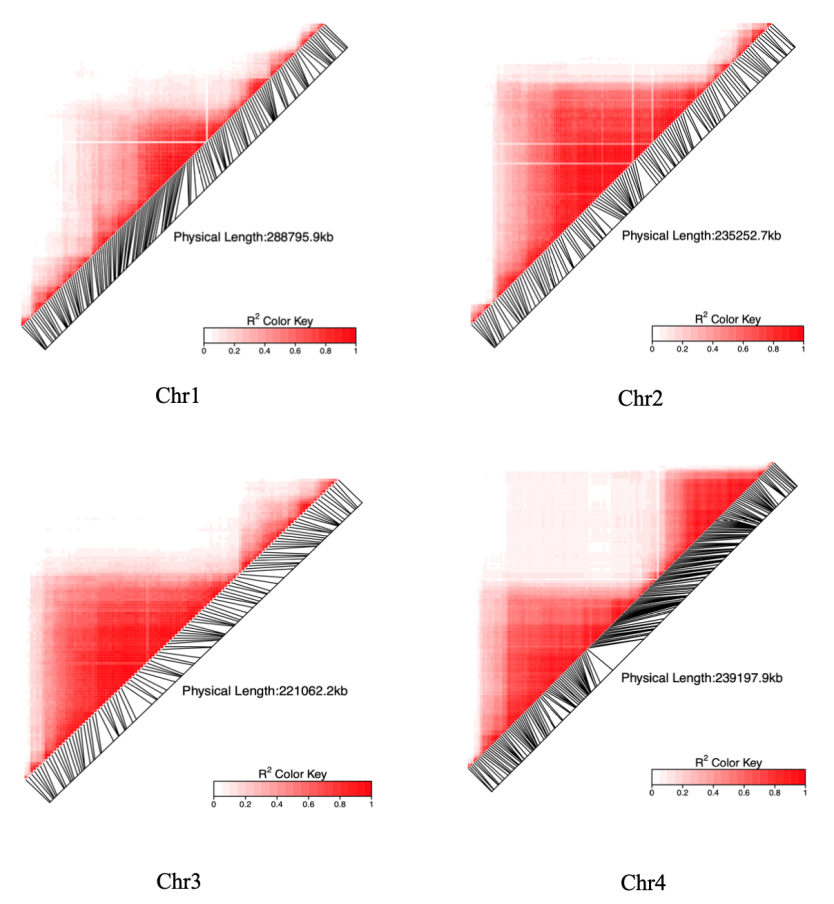


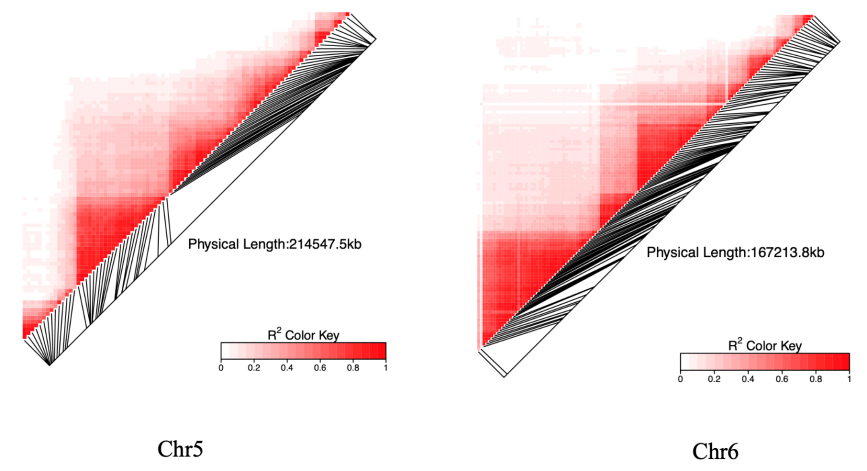


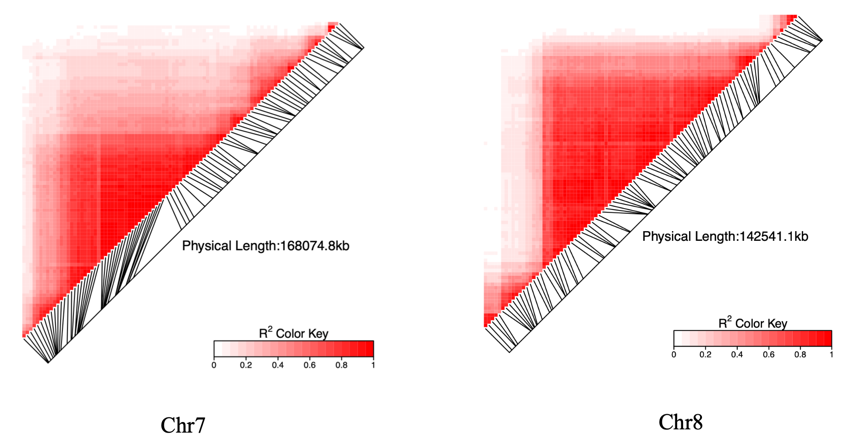


**
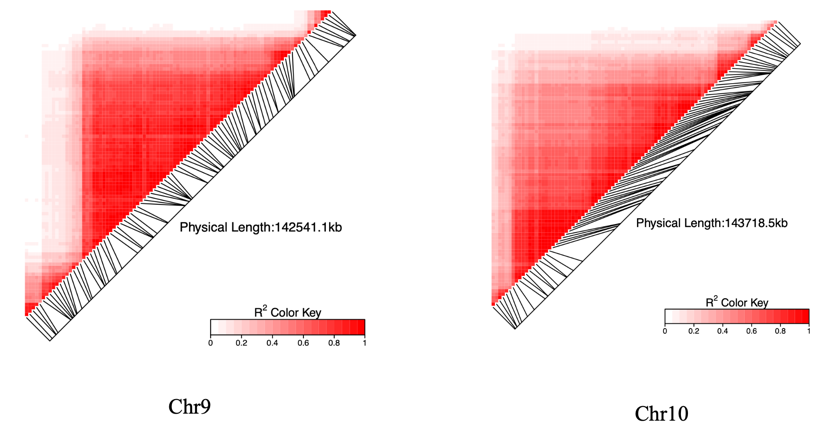
**

**Fig. S2** Linkage disequilibrium (LD) heat map of each linkage group. Color Key, LD.

**Table. S3** The number of SNP markers accepted as cofactors in the statistical model of QTL mapping

| Chromosome | Marker | Rank | F-Stat | DOF | Chromosome | Marker | Rank | F-Stat | DOF |
| --- | --- | --- | --- | --- | --- | --- | --- | --- | --- |
| 1 | 8 | 59 | 6.4041 | 81 | 3 | 90 | 38 | 6.506 | 102 |
| 1 | 9 | 52 | 4.0943 | 88 | 3 | 95 | 66 | 5.46 | 74 |
| 1 | 12 | 78 | 7.5037 | 62 | 3 | 97 | 9 | 4.981 | 131 |
| 1 | 13 | 51 | 5.7679 | 89 | 3 | 98 | 10 | 7.546 | 130 |
| 1 | 20 | 50 | 9.7781 | 90 | 3 | 101 | 6 | 4.636 | 134 |
| 1 | 22 | 90 | 5.6235 | 50 | 3 | 103 | 7 | 5.899 | 133 |
| 1 | 24 | 49 | 4.5983 | 91 | 4 | 80 | 39 | 5.201 | 101 |
| 1 | 29 | 62 | 5.1659 | 78 | 4 | 81 | 61 | 5.585 | 79 |
| 1 | 38 | 96 | 0.8748 | 44 | 4 | 82 | 55 | 4.917 | 85 |
| 1 | 41 | 70 | 5.9487 | 70 | 4 | 117 | 24 | 5.179 | 116 |
| 1 | 48 | 68 | 7.0161 | 72 | 4 | 121 | 31 | 6.361 | 109 |
| 1 | 96 | 16 | 6.3581 | 124 | 4 | 132 | 36 | 6.145 | 104 |
| 1 | 97 | 15 | 4.5012 | 125 | 4 | 134 | 88 | 6.594 | 52 |
| 1 | 100 | 64 | 6.3999 | 76 | 4 | 155 | 30 | 10.74 | 110 |
| 1 | 121 | 37 | 5.8025 | 103 | 4 | 156 | 25 | 6.994 | 115 |
| 1 | 123 | 100 | 1.5187 | 40 | 4 | 191 | 35 | 5.575 | 105 |
| 1 | 131 | 41 | 4.4752 | 99 | 4 | 192 | 34 | 4.68 | 106 |
| 1 | 152 | 81 | 5.9814 | 59 | 4 | 197 | 46 | 4.439 | 94 |
| 2 | 2 | 28 | 7.3084 | 112 | 4 | 200 | 26 | 6.385 | 114 |
| 2 | 4 | 54 | 5.173 | 86 | 5 | 10 | 3 | 5.272 | 137 |
| 2 | 6 | 27 | 6.5123 | 113 | 5 | 11 | 11 | 8.182 | 129 |
| 2 | 11 | 20 | 6.3214 | 120 | 5 | 12 | 18 | 5.201 | 122 |
| 2 | 16 | 23 | 5.7286 | 117 | 5 | 13 | 17 | 5.499 | 123 |
| 2 | 17 | 63 | 6.1954 | 77 | 5 | 28 | 77 | 4.488 | 63 |
| 2 | 22 | 19 | 5.773 | 121 | 5 | 83 | 67 | 6.306 | 73 |
| 2 | 28 | 48 | 4.7693 | 92 | 5 | 84 | 87 | 7.799 | 53 |
| 2 | 29 | 58 | 6.9555 | 82 | 6 | 9 | 40 | 5.068 | 100 |
| 2 | 30 | 56 | 6.5608 | 84 | 6 | 21 | 79 | 7.151 | 61 |
| 2 | 34 | 73 | 6.2189 | 67 | 7 | 31 | 74 | 4.994 | 66 |
| 2 | 37 | 32 | 5.7298 | 108 | 7 | 40 | 14 | 4.311 | 126 |
| 2 | 65 | 75 | 6.7268 | 65 | 7 | 55 | 80 | 5.165 | 60 |
| 2 | 91 | 72 | 5.1794 | 68 | 7 | 56 | 84 | 7.467 | 56 |
| 2 | 95 | 98 | 4.8094 | 42 | 7 | 57 | 94 | 5.401 | 46 |
| 2 | 101 | 57 | 8.2522 | 83 | 7 | 65 | 97 | 0.004 | 43 |
| 2 | 112 | 76 | 4.6033 | 64 | 7 | 77 | 45 | 5.06 | 95 |
| 2 | 113 | 92 | 5.1164 | 48 | 7 | 81 | 8 | 5.136 | 132 |
| 2 | 125 | 42 | 5.5539 | 98 | 7 | 88 | 2 | 5.809 | 138 |
| 3 | 1 | 43 | 4.5524 | 97 | 8 | 5 | 1 | 6.829 | 139 |
| 3 | 2 | 60 | 5.9285 | 80 | 8 | 8 | 5 | 4.37 | 135 |
| 3 | 3 | 47 | 4.1531 | 93 | 8 | 9 | 4 | 6.129 | 136 |
| 3 | 5 | 44 | 5.6444 | 96 | 9 | 18 | 82 | 5.146 | 58 |
| 3 | 29 | 91 | 4.0927 | 49 | 9 | 23 | 86 | 6.421 | 54 |
| 3 | 30 | 29 | 9.1096 | 111 | 9 | 26 | 93 | 7.057 | 47 |
| 3 | 33 | 21 | 5.7863 | 119 | 9 | 42 | 83 | 10.94 | 57 |
| 3 | 36 | 22 | 8.2231 | 118 | 9 | 71 | 95 | 6.083 | 45 |
| 3 | 37 | 71 | 6.3587 | 69 | 10 | 2 | 65 | 7.702 | 75 |
| 3 | 48 | 53 | 4.8885 | 87 | 10 | 4 | 13 | 6.439 | 127 |
| 3 | 52 | 89 | 5.615 | 51 | 10 | 6 | 12 | 4.523 | 128 |
| 3 | 67 | 99 | 1.7038 | 41 | 10 | 75 | 69 | 6.905 | 71 |
| 3 | 88 | 85 | 5.4825 | 55 | 10 | 87 | 33 | 6.16 | 107 |


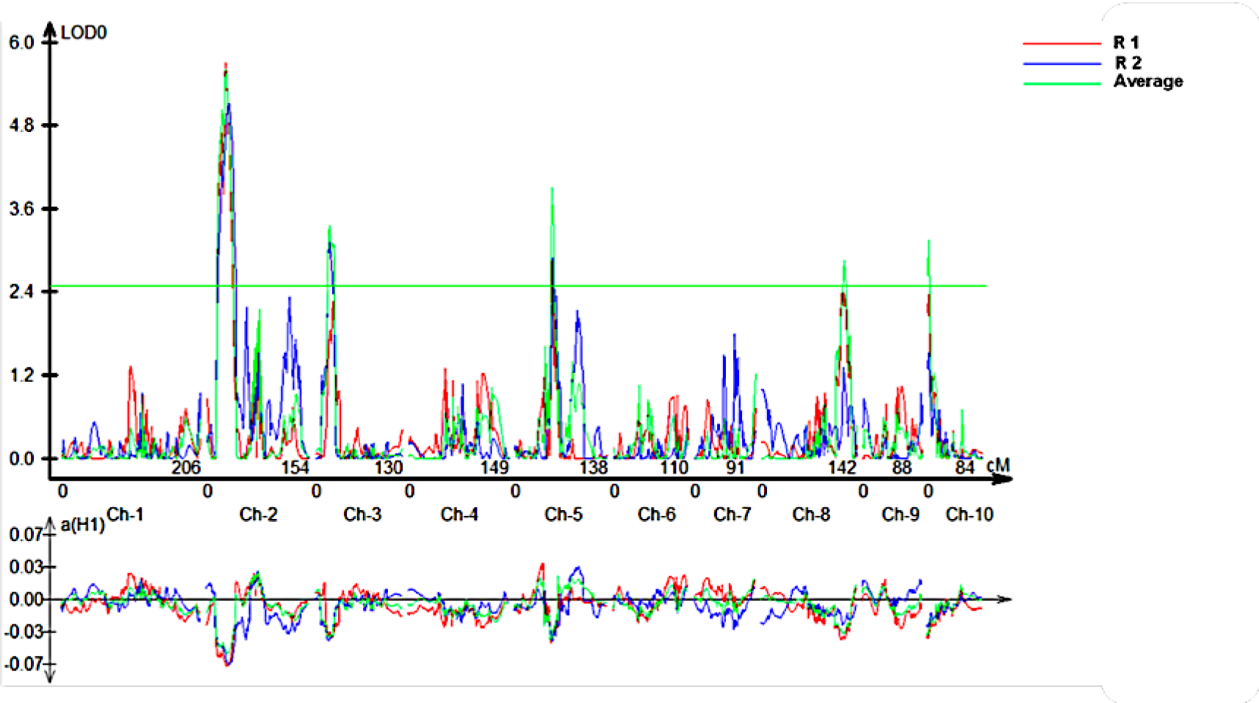


**Fig. S3** Detection of QTLs conferring resistance to MRDD. LOD profiles and the additive effect explained by QTLs of ten maize chromosomes. The legend with different colors to the right indicates the sources of the phenotypic data. *qMrdd2* was consistently detected in each field trial and averaged. R 1, R 2: repeat 1 and repeat 2 of DSI phenotypic data; Average: the average DSI phenotype of each RIL in repeat 1 and repeat 2.


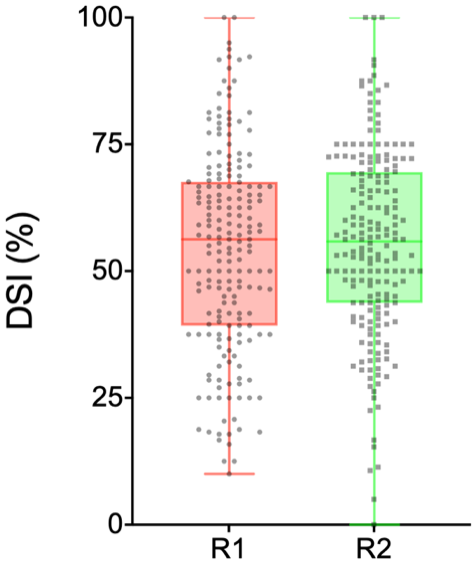


**Fig. S4** Boxplot graph of DSI values of the initial mapping population. R1, R2: repeat 1 and repeat 2 of DSI phenotypic data.

**Table S4** Markers developed to map the *qMrdd2* locus

| **Marker** | **Physical Location (bp)** | **Forward primer (5′-3′)** | **Reverse primer (5′-3′)** | **Annealing temp. (℃)** | **Type** |
| --- | --- | --- | --- | --- | --- |
| D184 | 1,830,602 | CTTGGTGCAGCAACTTCAGT | GTCTTAGCAGCCAATGGAGC | 60 | SSR |
| D387-2 | 3,876,416 | GATCGTGTGTGTGATGTGCA | GCTGCCTAAACGACGCATTA | 60 | SSR |
| D550 | 5,504,255 | CGGAGCCAAAGTTATCACCG | TTCCTCCGTCTCGTTACACC | 60 | SSR |
| D664 | 6,644,858 | CCTAAACCCGCCAACACAAA | GTTCTCTGATTCGGCTGCTG | 60 | SSR |
| D829-0 | 8,2901,74 | AGCTAGCTCAGTACTGGGGA | ATGTGTGAACAGCAGCCAAC | 60 | SSR |
| D936-2 | 9,372,421 | GATCAAGGTCGATGCATGGG | GTGGTGTGCGTGTAGTGTAC | 60 | SSR |
| D1116 | 10,710,944 | CACCAAGCTAGTCTAAGGGGT | CCACCCCTGATTTGACTGGA | 60 | SSR |
| D122-6 | 12,265,333 | TGCATCCGTTGTTTCGATCC | GATCGGTTCTTTGCTTGCCA | 60 | SSR |
| D1347 | 13,475,771 | CGAGTCGAATCATGCATGCA | CCAGACGTGCTATTTCCGTG | 60 | SSR |
| D1600 | 16,004,424 | ATGCCATGGGTTGCAATCTC | ATAGTGTAGACCCTGCTCGC | 60 | SSR |
| A1 | 8,457,905 | TGTGTTGACAGGACTCGTAGA | TGGAGTGTTGTTATGGGAGCT | 60 | InDel |
| A10 | 9,189,005 | CAACATTGCAAACCGTCGC | TGGTACATGTCACAGCCGT | 60 | InDel |
| S33 | 9,431,132 | CCGGCCTTTTATACACGCTC | GCTGTGCTCTTCCAAAGTCC | 60 | InDel |
| N37 | 9,538,355 | TGATGTGCCTGAATCATGGT | ACCCAAAGCCTCCTTCAGAT | 60 | InDel |
| N31 | 9,729,801 | AACGGTATCCCTGTGCTACA | TGTGCAGCTTCGTGTTTGAT | 60 | InDel |
| N42 | 10,306,610 | TCAGGCAGCGGATGAACA | CGGACCAAAGTGAGCCAAC | 60 | InDel |
| C2 | 11,829,589 | CTCTGCCGATTTGTGTGGTC | ATGTGGGTTGTGTCGATGAC | 60 | InDel |
| C3 | 11,998,554 | ATCGTTTTACCGGTCAGCCT | GCAACAACCGTCGAACCTAT | 60 | InDel |
| C6 | 12,060,557 | TGCCGACCTAAACATTCTTTCT | CCAAGAGCATGAACGTCACC | 60 | InDel |
| C13 | 12,108,811 | CGCACATGAGAAGAAGCCTC | GTGCATGGTGGTCAGATTCC | 60 | InDel |
| C18 | 12,171,245 | AGGTGCTCACTCATATCTCTCA | GTGATCAAAAGAACAACATACGC | 60 | InDel |

Physical Location (bp): the physical location of the QTL (APGv4).

**Table S5** Criteria applied to analysis SNP database

|  | **Criterion** |
| --- | --- |
| 1 | Delete SNPs with no polymorphism in both parents. |
| 2 | Delete SNPs whose parents are heterozygous. |
| 3 | Delete multiple copies of SNP (chr hit number > 1 in the annotation information) |
| 4 | Delete SNPs with missing rate is over 20% |
| 5 | Count the heterozygosity rate of each SNP (if the heterozygosity rate is high, this SNP may still be problematic) |
| 6 | Count the minimum allele frequency of each SNP, delete SNP with MAF less than 0.05 (theoretically 1:1) |

MAF: Minor Allele Frequency
